# Integration of Transcriptomics and Microbiomics Reveals the Responses of *Bellamya aeruginosa* to Toxic Cyanobacteria

**DOI:** 10.3390/toxins15020119

**Published:** 2023-02-01

**Authors:** Xianming Yang, Jinyong Zhu, Chaoyang Hu, Wen Yang, Zhongming Zheng

**Affiliations:** School of Marine Sciences, Ningbo University, Ningbo 315211, China

**Keywords:** *Bellamya aeruginosa*, transcriptome, intestinal microbiota, toxic cyanobacteria, microcystins

## Abstract

Frequent outbreaks of harmful cyanobacterial blooms and the cyanotoxins they produce not only seriously jeopardize the health of freshwater ecosystems but also directly affect the survival of aquatic organisms. In this study, the dynamic characteristics and response patterns of transcriptomes and gut microbiomes in gastropod *Bellamya aeruginosa* were investigated to explore the underlying response mechanisms to toxic cyanobacterial exposure. The results showed that toxic cyanobacteria exposure induced overall hepatopancreatic transcriptome changes. A total of 2128 differentially expressed genes were identified at different exposure stages, which were mainly related to antioxidation, immunity, and metabolism of energy substances. In the early phase (the first 7 days of exposure), the immune system may notably be the primary means of resistance to toxin stress, and it performs apoptosis to kill damaged cells. In the later phase (the last 7 days of exposure), oxidative stress and the degradation activities of exogenous substances play a dominant role, and nutrient substance metabolism provides energy to the body throughout the process. Microbiomic analysis showed that toxic cyanobacteria increased the diversity of gut microbiota, enhanced interactions between gut microbiota, and altered microbiota function. In addition, the changes in gut microbiota were correlated with the expression levels of antioxidant-, immune-, metabolic-related differentially expressed genes. These results provide a comprehensive understanding of gastropods and intestinal microbiota response to toxic cyanobacterial stress.

## 1. Introduction

In recent years, the influences of climate change and water eutrophication on aquatic ecosystems have received close attention globally due to frequent outbreaks of toxic cyanobacterial blooms [1]. Extreme proliferation of cyanobacteria not only severely disrupts the function of aquatic ecosystems, but release of algal toxics also increases the risk of water safety. The adverse environmental effects of one of the common bloom-forming cyanobacteria, *Microcystis aeruginosa*, and the toxins (microcystins, MCs) it produces are typical examples [2]. Exposure to MCs poses a health risk to aquatic animals, wild life, domestic animals, and humans upon drinking or ingesting toxic cyanobacteria in the water [3]. Aquatic organisms have been extensively studied because of their closer exposure to toxic cyanobacteria or MCs [4,5].

However, most of the existing studies on the effects of bloom-forming cyanobacteria and/or cyanotoxins on aquatic animals have focused on fish [6], shellfish [7], and crustaceans [8], whereas studies on freshwater gastropods are relatively insufficient. To date, a few reports on the response of freshwater snails to MCs have also been investigated from only a single perspective of bioaccumulation [9], biochemistry [10], histopathology [11], transcriptomics [12] and metabolomics [13]. For instance, the toxins in various organs and tissues of *Radix swinhoei* exposed to microcystin-leucine arginine (MC-LR) showed different distributions [14]. Exposure to toxic cyanobacteria resulted in damage to many organelles and an increase in the activities of phosphatases and glutathione S-transferases (GSTs) [10]. Toxic cyanobacteria have been shown to alter several gene expression levels in the freshwater snail *Parafossarulus striatulus* [12]. MCs exposure could cause hepatic energy expenditure and various metabolic disorders of energy substance [15]. Although omics approaches (e.g., genomics, transcriptomics, proteomics, and metabolomics) have proven to be a powerful tool to facilitate toxicological effects and risk assessment, thus making an important contribution to the understanding and unraveling of the molecular mechanisms of response to environmental stress [16], systematically and comprehensively analyzing the regulatory mechanisms of complex physiological processes using single omics are difficult, whereas the combined analysis of multi-omics could jointly explore the underlying regulatory network mechanisms in organisms and provide a more comprehensive examination of the response mechanisms of organisms. Several studies using a multi-omics approach have provided comprehensive insights into the molecular mechanisms of organic pollutants (e.g., perfluorooctane sulfonate and imidacloprid) at the molecular level [17,18].

The gut has long been considered one of the most important organs of microbe/host interactions. The gut provides a barrier for host health that consists of intestinal mucosa and normal parasitic microbiota. Animal bacteria contribute to their hosts’ health in terms of nutrition, immunity, digestion, and development [19]. This finding has also been demonstrated in snails, where intestinal bacteria are important contributors to many physiological processes [20]. In particular, gut microbes help their snail host digest plant-derived carbohydrates [21]. Different reproductive strategies and the transition of sexuality of the same snail species could be associated with microbial symbionts [22]. Some intestinal microbes that adhered to gut mucosa could stimulate immune cells response via Toll-like receptors [23]. The microbiota is highly plastic, and it could respond rapidly to changes in host diet or environmental conditions by altering community composition, mutating, and exchanging genetic material with bacteria in the environment [24]. Hence, the gut microbiota is an important reason for the rapid adaptation of the host to drastic environmental changes, which needs to be further investigated in snails exposed to toxic cyanobacteria.

*Bellamya aeruginosa* is one of the most important benthic animals in freshwater ecosystems, and it is widely distributed along the littoral zones of rivers and lakes in East Asia and indeed all over the world [25,26,27]. Moreover, *B. aeruginosa* is also recognized as a sentinel organism to bio-monitor and assess ecological risk of environmental contamination [28]. Evidence revealed that *B. aeruginosa*, as a facultative suspension feeder, has the potential for biological management of cyanobacterial blooms [29]. Given the practical significance and importance of *B. aeruginosa*, comprehensive research is urgently needed on the response of snails to toxic cyanobacteria. Therefore, this study revealed the dynamics and response patterns of transcriptome and gut microbiome in snails during toxic cyanobacterial exposure. The relationship between the differentially expressed genes (DEGs) and intestinal flora in snails exposed to toxic cyanobacteria was also explored. This study could provide comprehensive information to elucidate the potential mechanisms of snail response to toxic cyanobacteria.

## 2. Results

### 2.1. Snail Mortality

**Figure 1:**
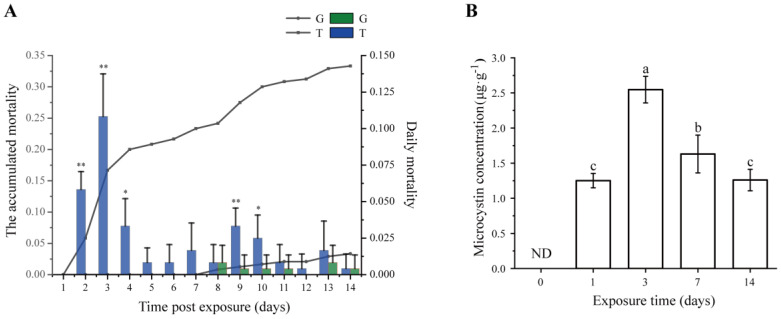
(**A**) Mortality and (**B**) microcystin concentration in *Bellamya aeruginosa* during the 14-day exposure test. The green and blue bars indicate the daily mortality rates of the control group (G) and treatment group (T), respectively. Lines with dots and squares indicate the accumulated mortality of the G group and T group, respectively. Error bars indicate the mean ± SD (*n* = 6). Asterisks represent significant difference (* *p* < 0.05; ** *p* < 0.01). A significant difference was found between the different timepoints in the T group by different small letters (*p* < 0.05). ND, not determined.

During a 14-day exposure, the mortality of snails was recorded. As shown in Figure 1A, at days 2–4 and 9–10 after exposure to toxic cyanobacteria, the daily mortality significantly increased (*t*-test, *p* < 0.05), and the highest daily mortality occurred at day 3. No snail died in the control group (G group) until 7 days post exposure, after which a small number of snails died (3.33%, about one snail per tank), which may be due to the normal mortality phenomenon. However, the accumulated mortality reached 33.3% at 14 days post exposure in the treatment group (T group).

### 2.2. Dynamics of MC Concentration

The dynamics of MC concentrations that bioaccumulated in the hepatopancreas for a period of 14-day exposure are shown in Figure 1B. MCs were not detected in the G group throughout the entire exposure experiment. The MC content in the T group increased steeply to 1.25 ± 0.10 μg·g^−1^ dry weight (DW) at day 1, and kept increasing to the highest MC content of 2.55 ± 0.19 μg·g^−1^ DW at day 3. A notable detail is that the accumulative MCs significantly decreased (ANOVA test, *p* < 0.05) at days 7 and 14 compared with those at day 3, suggesting that the mechanism of detoxification of *B. aeruginosa* was at work.

### 2.3. Transcriptomic Analysis of Hepatopancreas

cDNA libraries were constructed from the mRNA extracted from the hepatopancreas of 24 samples (two treatments: control and T group; four timepoints; and three biological replicates). A total of 12.42 Gb clean reads were yielded in the construction of reference transcriptome, and 94,781 unigenes (hereafter genes) were obtained, with total length, average length, N50, and GC content of 66,260,357 bp, 699 bp, 1092 bp, and 36.01%, respectively. All genes were annotated in accordance with the seven main functional databases: 26,437 (NR: 27.89%), 12,244 (NT: 12.92%), 16,928 (SwissProt: 17.86%), 16,678 [Gene Ontology (GO): 17.60%], 15,294 (KOG: 16.14%), 17,362 (Pfam: 18.32%), and 18,511 [Kyoto Encyclopedia of Genes and Genomes (KEGG): 19.53%]. A total of 34,002 (35.87%) genes were effectively matched using at least one database, and 2654 genes were matched in all seven databases. Among the 26,437 genes matched to the NR database, 66.7% were similar to *Pomacea canaliculata*, followed by *Aplysia californica* (2.0%), *Lottia gigantea* (1.3%), *Elysia chlorotica* (1.3%), and *Biomphalaria glabrata* (1.2%). The total clean reads from 24 libraries were mapped to the reference database, and the results are shown in Table S1.

**Figure 2:**
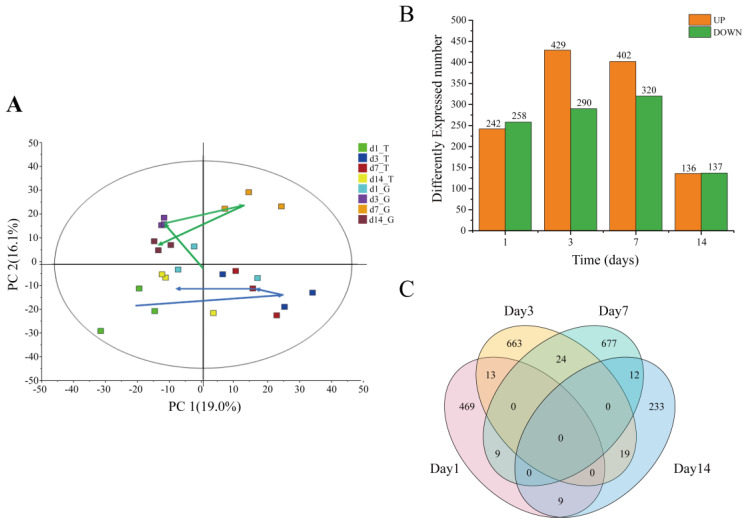
Overview of transcriptome changes in *B. aeruginosa* exposed to toxic cyanobacteria. (**A**) Principal component analysis of transcriptome of *B. aeruginosa*. The green and blue arrows indicate temporal trends for the control group (G) and treatment group (T), respectively. The horizontal and vertical axes represent the first and second principle components, respectively; (**B**) numbers of significantly URGs and DRGs in *B. aeruginosa* at days 1, 3, 7, and 14; (**C**) Venn diagram showing the comparison of the number of DEGs for the two groups at days 1, 3, 7, and 14.

Principal component analysis (PCA) showed that the samples in the G group at days 1, 3, and 14 were gathered, indicating a relatively similar transcriptome in this group. A distinct expression profile between day 3 or 7 and day 1 or 14 was observed in T group. The gene expression on days 1 and 14 in the T group was notably more similar to that in the G group (Figure 2A). Among all expressed genes, 2128 genes were differentially expressed between the T group and the G group (Table S2). More up-regulated genes (URGs) were observed than down-regulated genes (DRGs) on days 3 and 7, whereas the number of URGs and DRGs was about the same on days 1 and 14. The distribution of DEGs showed that both genes increased and then decreased with time (Figure 2B). Moreover, a number of both DEGs were co-regulated at two timepoints, whereas no shared DEG was expressed at more than two timepoints (Figure 2C).

#### 2.3.1. GO Functional Annotation of DEGs

GO functional enrichment analysis was performed at days 1, 3, 7, and 14 to understand the biological processes that could be differentially regulated by toxic cyanobacteria. The analysis showed that 121, 147, 216, and 72 DEGs could be classified into 21, 22, 21, and 21 subcategories at days 1, 3, 7, and 14, respectively (Figure S1). In general, the main subcategories of GO term (top five in level 2) were cellular anatomical entity, binding, catalytic activity, cellular process, and metabolic process. For biological processes, cellular process, metabolic process, and biological regulation were the most significantly enriched terms. A notable detail is that the immune system process and response to stimulus were enriched in the metabolic process. However, antioxidant activity was significantly enriched at days 7 and 14, which indicates that the antioxidant-related DEGs were more significant at day 7.

#### 2.3.2. KEGG Pathway Enrichment Analysis of DEGs

KEGG pathway enrichment analysis was further performed to identify sets of DEGs involved in specific biological functions. The URGs and DRGs were subjected to KEGG enrichment analysis to reveal the mechanism of resistance to toxic cyanobacteria between the T group and the control. A KEGG pathway was considered significantly enriched if the *p* value was <0.05.

**Figure 3:**
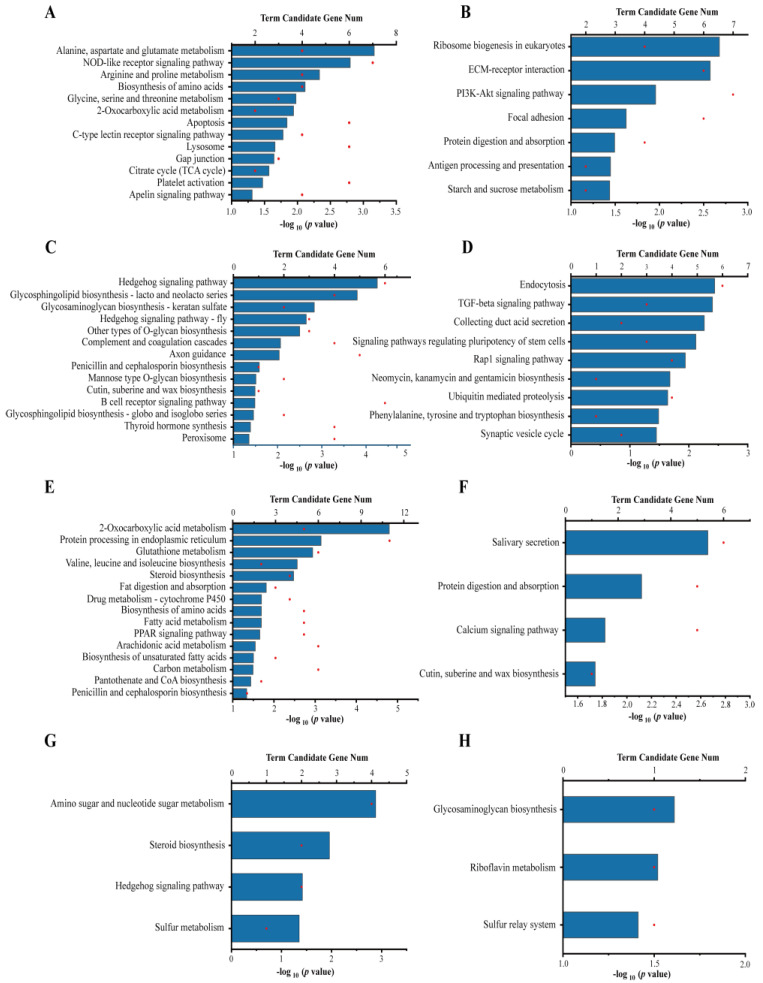
Enriched KEGG pathways of DEGs. (**A**,**C**,**E**,**G**) Enriched KEGG pathways of URGs at day 1, 3, 7, and 14, respectively. (**B**,**D**,**F**,**H**) Enriched KEGG pathways of DRGs at day 1, 3, 7, and 14, respectively. The degree of KEGG pathway enrichment is represented by the *p* value. The red dots represent the number of genes enriched in the KEGG pathway.

At day 1, 20 pathways were significantly enriched in the KEGG database, including 13 for URGs and seven for DRGs. Among the up-regulated pathways, metabolism was the most dominant category, and it included several subcategories: carbohydrate metabolism and energy metabolism, such as citrate cycle (TCA cycle) and 2-oxocarboxylic acid metabolism, and amino acid metabolism and synthesis, such as alanine, aspartate and glutamate metabolism; arginine and proline metabolism; and glycine, serine and threonine metabolism. Immune-related pathways were also significantly enriched, including NOD-like receptor signaling pathway, apoptosis, C-type lectin receptor signaling pathway, lysosome, and platelet activation (Figure 3A). The top three pathways significantly enriched for DRGs were ribosome biogenesis in eukaryotes, ECM-receptor interaction, and PI3K-Akt signaling pathway (Figure 3B).

At day 3, the URGs were significantly enriched in 14 pathways, including immune-related pathways, such as complement and coagulation cascades and B cell receptor signaling pathway; and some glycan biosynthesis and metabolism pathways (Figure 3C). The top three significantly enriched pathways for DRGs were endocytosis, TGF-beta signaling pathway, and collecting duct acid secretion (Figure 3D).

At day 7, the URGs were enriched in 15 pathways, including carbohydrate metabolism and energy metabolism, such as 2-oxocarboxylic acid metabolism, carbon metabolism, and pantothenate and CoA biosynthesis; lipid metabolism, such as steroid biosynthesis, fat digestion and absorption, fatty acid metabolism, PPAR signaling pathway, arachidonic acid metabolism, and biosynthesis of unsaturated fatty acids; and amino acid metabolism and protein biosynthesis, such as valine, leucine and isoleucine biosynthesis, and biosynthesis of amino acids. A notable detail is that several pathways related to stress response and exogenous biodegradation were also significantly enriched, such as glutathione metabolism and drug metabolism (cytochrome P450), indicating a significant increase in oxidative stress and exogenous degradation activity at day 7 (Figure 3E). The top three pathways significantly enriched for DRGs were salivary secretion, protein digestion and absorption, and calcium signaling pathway (Figure 3F).

At day 14, the URGs were significantly enriched in the pathways related to carbohydrate metabolism and energy metabolism, including sulfur metabolism, amino sugar and nucleotide sugar metabolism, and lipid metabolism, such as steroid biosynthesis (Figure 3G). The only three significantly enriched pathways for DRGs were glycosaminoglycan biosynthesis, riboflavin metabolism, and sulfur relay system (Figure 3H).

#### 2.3.3. Identification of Host Health-Related DEGs

Based on the NR database annotation, several DEGs were implicated in stress response, immune system, and metabolism, thereby providing an enhanced understanding of the host health status of snails induced by toxic cyanobacteria. Specifically, 36 DEGs, including stress response (8), immune (10), and metabolism (18), were mainly shared in at least two timepoints, as displayed in Table 1.

#### 2.3.4. Validation of Gene Expression in the Transcriptome

**Figure 4:**
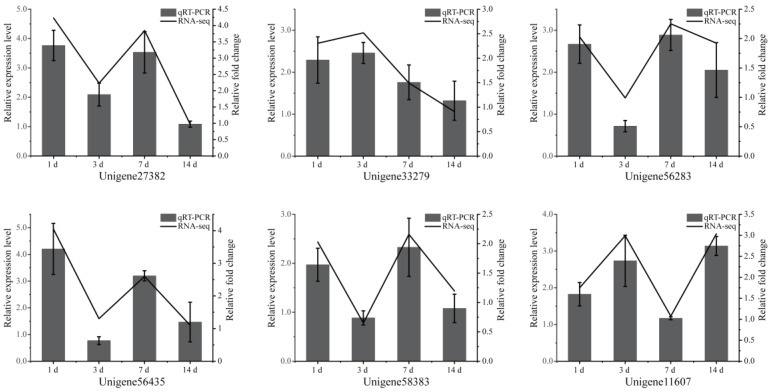
Changes in expression of six DEGs after exposure to toxic cyanobacteria, as revealed by qRT-PCR. The left and right vertical axes refer to the relative expression measured by qRT-PCR and the RNA-seq expression, respectively. The values of qRT-PCR are expressed as mean ± SD (*n* = 3).

Six DEGs were randomly selected and identified by qRT-PCR to evaluate the reliability of the transcriptome data. The results showed that the expression tendency of qRT-PCR matched that of RNA-seq (Figure 4), indicating that the experiment of RNA sequencing analysis was reliable.

### 2.4. Intestinal Microbiota Analysis

#### 2.4.1. Effects of Toxic Cyanobacteria on the Intestinal Microbiota of *B. aeruginosa*

**Figure 5:**
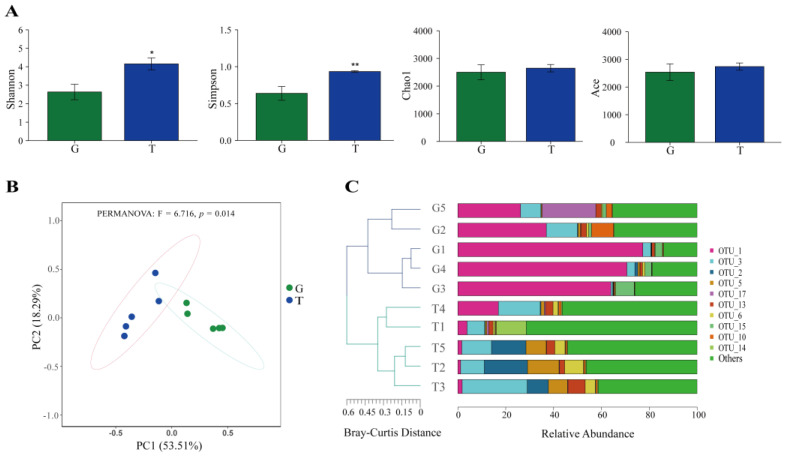
Microbiome alterations induced by toxic cyanobacterial exposure. (**A**) Alpha diversity; (**B**) principal coordinate analysis (PCoA); (**C**) hierarchical clustering tree based on the Bray–Curtis method at OTU level. * *p* < 0.05, ** *p* < 0.01.

A total of 2512 operational taxonomic units (OTUs), 38 phyla, 86 classes, 174 orders, and 370 genera were detected in the whole samples. Among these OTUs, 409 (16.3%) and 705 (28.1%) were exclusive in samples from the G and T groups, and 1398 (55.7%) were shared by the two groups (Figure S2). The composition of the intestinal microbiota of *B. aeruginosa* was altered after 14 days of toxic cyanobacterial exposure. The Shannon and Simpson indices in the T group increased compared with those in the G group, whereas the Chao1 and Ace indices of microbial community did not significantly change (Figure 5A). However, the results of analysis of similarity (ANOSIM) showed that the intestinal microbial community composition was significantly different between the two groups (*R* > 0, *p* < 0.01, Figure S3). The principal coordinate analysis (PCoA) results also showed that the microbial composition of the T group was notably different from that of the G group (PERMANOVA results: F = 6.716, *p* = 0.014; Figure 5B). Hierarchical clustering analysis (HCA) could separate the two groups based on the Bray–Curtis method at the OTU level (Figure 5C).

Exposure to toxic cyanobacteria changed the percentages of gut microbiota at the phylum and genus levels. The phyla Campilobacterota, Firmicutes, and Myxococcota in the T group increased compared with those in the G group, but those of Cyanobacteria, Fusobacteriota, and Halobacterota decreased (*p* < 0.05, Table S3). At the genus level, the relative abundances of *Chryseobacterium*, *Sphingobium*, *Corynebacterium*, *Thermomonas*, *Sulfurospirillum*, *Aquimonas*, and *Mycoplasma* in the T group increased, whereas those of *Cetobacterium*, *Tundrisphaera*, *Bacteroides*, *Candidatus_Rhabdochlamydia*, and *Pseudorhodobacter* decreased (*p* < 0.05, Table S4).

#### 2.4.2. Co-Occurrence Networks

A microbial co-occurrence network was constructed at the phylum level to compare the co-occurrence patterns of different groups of microbial communities (Figure S4). The T group showed a microbial network with more complex structure than the G group. For example, the T group had a higher number of network nodes, edges, average connectivity (avgK), and average clustering coefficient (avgCC) than the G group. A notable detail is that the occupancy of the negative correlations in the T group was elevated (Table 2).

#### 2.4.3. Functional Prediction Analysis of Intestinal Microbiota

**Figure 6:**
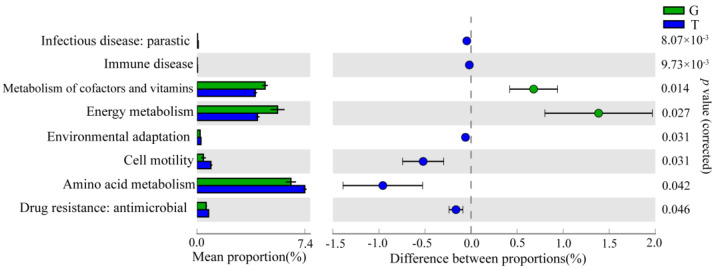
KEGG function prediction of intestinal bacterial communities at level 2. The middle part shows the difference between the proportions of functional abundance within the 95% confidence interval, and the value in the rightmost is the *p* value.

PICRUSt2 analysis was used to perform KEGG functional prediction to characterize the changes in the intestinal microbial function of *B. aeruginosa* after MCs exposure. Based on the prediction of intestinal microbial function, compared with the G group, the T group had significant differences in “Infectious disease: parasitic,” “immune disease,” “metabolism of cofactors and vitamins,” “energy metabolism,” “environmental adaptation,” “cell motility,” “amino acid metabolism,” and “drug resistance: antimicrobial” at KEGG level 2 (Figure 6). At level 3, compared with the G group, many pathways were enriched, such as “porphyrin and chlorophyll metabolism;” “insulin signaling pathway;” “pentose and glucuronate interconversions;” “primary immunodeficiency;” and “glycine, serine, and threonine metabolism” (Table S5).

### 2.5. Association between the Intestinal Microbiota and the DEGs Related to Host Health

**Figure 7:**
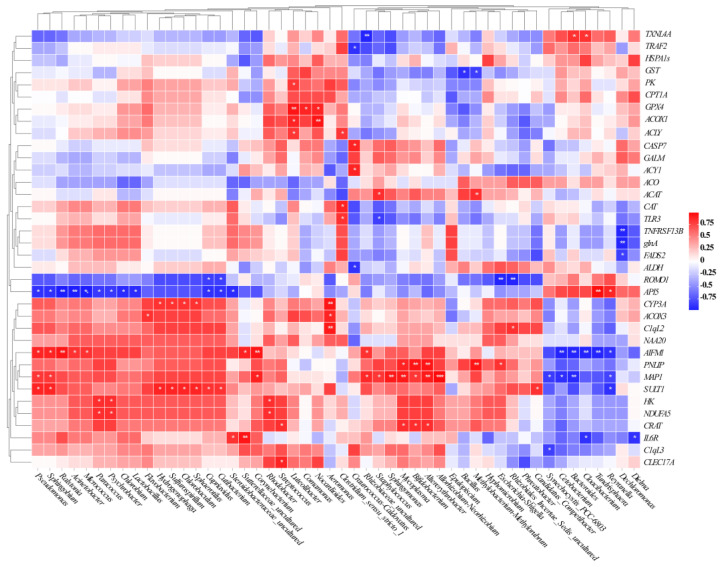
Spearman’s correlation analysis between host health-related DEGs and the genus level of intestinal microbiota. Red and blue squares represent positive and negative correlations, respectively. (* *p* < 0.05; ** *p* < 0.01; *** *p* < 0.001).

A heatmap was generated using Spearman’s coefficient test to reveal the relationships between the intestinal microbiota and gene expressions involved in host health. In the correlation between intestinal bacteria and host stress-response DEGs, *Pseudomonas*, *Sphingobium*, *Ralstonia*, *Acinetobacter*, *Morganella*, and *Lactobacillus* were positively correlated with changes in *SULT1* and *CYP3A*. Meanwhile, *Bacillus*, *Methylobacterium*, and *Staphylococcus* were negatively correlated with changes in *GST*, *TXNL4A*, and *GPX4*. In the correlation between intestinal bacteria and host immune-related DEGs, *Pseudomonas*, *Sphingobium*, *Ralstonia*, and *Acinetobacter* were positively correlated with changes in *AIFM1*, *C1qL2*, *IL6R*, *C1qL3*, *TLR3*, and *TNFRSF13B*, and negatively correlated with changes in *API5*. In the correlation between intestinal bacteria and host metabolism-related DEGs, *Staphylococcus*, *Sphingomonas*, *Mycoplasma*, *Bifidobacterium*, *Altererythrobacter*, and *Allorhizobium* were positively correlated with changes in *MAP1*, *PNLP*, and *CRAT*; *Synechocystis_PCC-6803*, *Cetobacterium*, and *Bacteroides* were negatively correlated with changes in *MAP1* and *PNLIP* (Figure 7).

## 3. Discussion

The bioaccumulation and distribution of MCs in gastropods, whether exposed in the field [30] or experimentally [31], have been widely reported, and the highest accumulated organ is the hepatopancreas [32]. In our case, *Bellamya* accumulated a large amount of MCs at the beginning of the exposure test and decreased at a later stage, similar to the results of previous studies [10,33]. Snail mortality in the toxic cyanobacterial group showed a similar trend, with the maximum daily mortality of snails being reached at the peak of toxin accumulation and decreasing with the reduction in toxin. A dose effect appeared to be present between mortality and MC accumulation, which agrees well with the findings of zebrafish exposed to MC-LR [34]. The reduction in toxin accumulation and mortality in the later stages of the experiment may be a specific manifestation of the adaptation of *Bellamya* to environmental stress.

### 3.1. Hepatopancreatic Transcriptomic Response to Toxic Cyanobacteria

Similar to previous studies, toxic cyanobacteria could alter the normal transcriptome of gastropods [12]. However, the difference is that the transcriptome of snails in this experiment had a tendency to recover. For example, the gene expression patterns of *Bellamya* at day 14 of exposure to toxic cyanobacteria was closer to that of the control group (Figure 2A), and the numbers of DEGs at day 14 was the lowest (Figure 2C). In addition, the numbers of DEGs (Figure 2C) and KEGG pathways (Figure 3) enriched for these DEGs were somewhat proportional to the accumulated concentrations of MCs. These results suggested that MCs may have the most predominant effect on the transcriptome of snails in a MC concentration-dependent manner, which is in accordance with recent studies of *Litopenaeus vannamei* to imidacloprid exposure [18]. In general, differential gene expression analysis demonstrated that toxic cyanobacterial exposure produced marked transcriptional changes, primarily related to stress response and xenobiotics biodegradation, immune function, and metabolic disorders. Similar results were also obtained in the toxicity studies of pure MCs in aquatic animals: purified MCs could exert detrimental effects on the immune system [35], metabolic activities [36], apoptosis [37], and redox disorder [38].

#### 3.1.1. Stress Responses and Xenobiotics Biodegradation

MCs could induce oxidative damage in animals and cause an increase in reactive oxygen species (ROS), leading to oxidative stress [39]. Activation of the Nrf2-ARE pathway could induce the expression of antioxidant enzymes that scavenge excess ROS and exert cytoprotective effects [40]. Nrf2, an essential transcription factor, was significantly up-regulated after exposure to toxic cyanobacteria for 7 days. Many downstream genes of this pathway, including the genes encoding for glutathione S-transferase (GST), catalase (CAT), superoxide dismutase, glutathione peroxidase, and ATP-binding cassette (ABC) transporters, also showed significant up-regulation. GST is one of phase II metabolic conjugation enzymes that could mediate the formation of MC-LR-GSH, which was seen as the first step in the detoxification of MCs [41]. In line with the results of the present study, GST was induced in *B. aeruginosa* exposed to *M. aeruginosa* [10]. ABC transporters have the ability to transport MC-LR-GSH conjugates, which are unable to diffuse through the membrane out of cells [42]. A significant up-regulation of the drug metabolism-cytochrome P450 pathway was notably observed at day 7. Cytochrome P450 (CYP 450) is a phase I metabolic enzyme involved in detoxification. The expression of gene encoding for CYP 450 was up-regulated significantly, which indicated the formation of the first line of detoxification defense [18].

#### 3.1.2. Immune Responses

The immune system is very sensitive to toxic cyanobacteria, and many studies have demonstrated the immunotoxicity of MCs in aquatic animals [43,44]. Gastropods, similar to other invertebrates, mainly rely on a non-specific intrinsic immune system to cope with environmental stresses [45]. In the present investigation, many significantly enriched KEGG pathways for URGs were related to the immune system at days 1 and 3 (Figure 3). Meanwhile, the expression levels of genes encoding several critical immune defense factors broadly described in other gastropods were induced to varying degrees in the T group [46]. Activation of the complement system, such as C1q, allows for efficient translocation and clearance of immune complexes and apoptotic vesicles, which are essential for maintaining a normal immune response in the body [47]. C-type lectin plays an important role as pattern recognition receptors in the regulation of the body’s immunity against microbial infection [48]. Cathepsins play an important role in innate and acquired immunity [49]. TNF-induced apoptosis plays a vital role in the pathogenesis of inflammatory diseases. Apoptosis plays a crucial role in the removal of unwanted or abnormal cells [50]. The up-regulation of the apoptotic pathway may indicate that the body self-regulates and repairs cells damaged by MCs exposure, as evidenced by the induction of the expression levels of AIF and caspase 3 at days 1 and 3. In addition, some mRNAs encoding for proteins (API, Bcl2, and Bax inhibitor) involved in the inhibition of apoptosis were significantly up-regulated at days 7 and 14. These results suggested that cells tend to stabilize without the need for apoptosis mediation at a later stage.

#### 3.1.3. Energy Metabolism

Toxic cyanobacteria and MCs could induce hepatopancreatic energy depletion, accompanied by disorders in the metabolism of energy substances [13]. Many carbohydrate metabolic pathways were significantly enriched for the URGs. Carbohydrates are the main and direct source of energy for snails in response to environmental stress [51]. Many amino-acid metabolic pathways were significantly enriched for URGs at day 1. However, the biosynthetic pathways of branched-chain amino acids (BCAAs) were significantly up-regulated at day 7. The enhanced amino acid metabolism indicated an increased energy requirement of the body, and the enhanced synthesis may be a compensatory effect of BCAAs depletion. Lipid metabolism-related pathways were significantly enriched for URGs at days 3 and 7. Similar to these results, oral administration of MC-LR to mice induced disturbances in hepatic lipid metabolism [52].

### 3.2. Intestinal Microbiota in Response to Toxic Cyanobacterial Stress

The intestinal microbial diversity of snails was significantly increased when exposed to toxic cyanobacteria (*p* < 0.05; Figure 5A). These results are similar to those reported by Duan et al. [53]. However, this finding is contrary to the results of the previous field investigation [30], which may be related to the complex and variable environmental conditions in the field. Toxic cyanobacterial exposure also altered the community composition of gut microorganisms, increasing the relative abundance of Firmicutes (Table S3). In general, Proteobacteria, Bacteroidota, and Firmicutes were the three most abundant phyla in the intestinal microbiota of freshwater snails [30], generally consistent with the results in this paper. Members of the phylum Firmicutes are thought to regulate host energy metabolism by producing short-chain fatty acids [54]. In the T group, the abundance of some genera with the ability to degrade MCs increased, such as *Pseudomonas*, *Lactobacillus*, and *Ralstonia* (Table S4), in line with the results of the field experiment that showed the abundance of *Ralstonia* and *Pseudomonadales* increased after cyanobacterial bloom outbreak [30]. These results may suggest that toxic cyanobacterial stress makes the MCs-degrading flora more competitive and other microbiota less competitive. *Pseudomonas* could lyse *M. aeruginosa* cells and degrade the MCs produced by them [55]. *Lactobacillus* exopolysaccharides could be used as a nutritional or therapeutic agent to regulate the host’s immune system [56]. *Ralstonia* could secrete a variety of plant cell-wall degrading enzymes, and increased levels of these enzymes help the host adapt to changes in food sources [57]. The increased resistance of *B. aeruginosa* may be due to the fact that bacteria with protective properties against the host are more dominant after toxic cyanobacterial exposure.

The network of co-occurring gut microbiota showed higher edge, node number, and average connectivity (avgK) after toxic cyanobacterial exposure, suggesting stronger network interactions and making the intestinal microbes more closely related. The findings could be supported by the study of shrimp gut microbiota [58]. Complex networks that are better connected are more resistant to external disturbances than simple networks with poor connection [59]. One possible explanation for this finding is that the host disease creates an adaptive advantage, i.e., an empty ecological niche, for key taxa, thus outperforming other symbiotic flora to colonize the gut of the morbid snail [60]. Furthermore, the formation of such convergent and complex symbiotic patterns may result from adaptive drivers (i.e., host stress responses) affecting key taxa. Moreover, gut microbial metabolic pathways were altered, such as metabolisms of cofactors and vitamins, and energy metabolism pathways were down-regulated; amino acid metabolism, and immune disease pathways were up-regulated in the T group. We speculated that the altered structure and function of the gut microbial community may be a positive response to stress induced by toxic cyanobacterial exposure.

### 3.3. Relationship between Gut Microbial and Host Health-Related DEGs

Evidence showed that intestinal microorganisms have an important effect on the function of extraintestinal organs, such as the liver [61]. The liver is an important immune organ that could be exposed to bacteria of intestinal origin or bacterial metabolites, hormones, and neuromediators through the enterohepatic circulation [62]. The increased levels of *Pseudomonas*, *Sphingobium*, *Ralstonia*, *Morganella*, and *Lactobacillus* were positively correlated with changes in stress-response-related DEGs (*SULT1* and *CYP3A*), whereas *Bacillus*, *Methylobacterium*, and *Staphylococcus* were negatively correlated with changes in *GST*, *TXNL4A*, and *GPX4*, indicating that these bacteria may affect the stress response of *B. aeruginosa*. *Bacillus* and *Lactobacillus* are now widely used as probiotics that provide protective immunity, and they have been shown to be associated with *GST* expression [63,64]. *Pseudomonas*, *Sphingobium*, *Ralstonia*, and *Acinetobacter* were positively correlated with changes in *AIFM1*, *C1qL2*, *IL6R*, *C1qL3*, *TLR3*, and *TNFRSF13B*, while *Staphylococcal* was negatively correlated with changes in *TLR3*. TLRs play a crucial role in mediating the host’s response to *Staphylococcal* [65]. There was evidence that Pyocyanin production by *Pseudomonas* induced neutrophil apoptosis in human tissue and had an effect on cytokines (IL-6), which was similar to our results [66]. *Staphylococcus*, *Sphingomonas*, *Mycoplasma*, *Bifidobacterium*, *Altererythrobacter*, and *Allorhizobium* were positively correlated with changes in *MAP1*, *PNLIP*, and *CRAT*, indicating that these bacteria may affect the amino acid and lipid metabolism of the host. Consistently, zhang et al. have revealed that intestinal microbiota could influence chicken growth by regulating fat metabolism, and *Sphingomonas* level was positively correlated with fat metabolism [67]. Additionally, the carotenoids produced by *Sphingomonas* bear antioxidant properties, and they could scavenge free radicals produced in the body [68]. *Reyranella*, *Cetobacterium*, and *Bacteroides* were negatively correlated with changes in *MAP1*. *Bacteroides* spp. could promote BCAA catabolism in brown fat and inhibit obesity [69]. The transcriptome results of the present study also revealed similar findings.

## 4. Conclusions

The response mechanism of *B. aeruginosa* subjected to toxic cyanobacterial exposure was investigated using integrated analysis of the transcriptome and microbiome. A total of 2128 DEGs were identified at different exposure periods. The GO enrichment analysis showed that the DEGs were significantly enriched in the cellular anatomical entity, binding, catalytic activity, cellular process, and metabolic process. The KEGG pathway analysis showed that immune-related pathways, such as the NOD-like receptor signaling pathway and apoptosis, were significantly induced at the first 7 days of toxic cyanobacteria exposure. The pathways associated with oxidative stress and detoxification were significantly enriched at the last 7 days of toxic cyanobacteria exposure. The microbiome results revealed that toxic cyanobacterial exposure altered the microbial composition, especially the enrichment of MC-degrading bacteria (*Pseudomonas*, *Lactobacillus*, and *Ralstonia*), and increased the diversity of gut microbiota. Meanwhile, the gut bacterial network complexity and function were also changed. The relation analysis found that the changes in gut microbiota were correlated with the expressions of antioxidant-, immune-, and metabolic-related DEGs. Collectively, this research provided a reference dataset for further investigation of gastropods’ response to toxic cyanobacteria and enriched the interactions between the gastropods and their gut microorganisms with toxic cyanobacteria.

## 5. Materials and Methods

### 5.1. Test Snail and Algae Cultivation

Snails (15 ± 3 mm shell length) were collected in a natural water body (Yinzhou Wetland Park, Ningbo, China) without cyanobacterial blooms. Snails with intact shells, uniform size, and good vigor were selected, and the periphyton and dirt of shells were removed. The unicellular green algae *Chlorella vulgaris* (strain NMBlud2006-2, provided by the Aquatic Ecology Laboratory of Ningbo University, Ningbo, China) were cultured in NMB3 medium (100 mg/L KNO_3_, 10 mg/L KH_2_PO_4_, 2.5 mg/L FeSO_4_·5H_2_O, 6 μg/L VB_1_, and 0.05 μg/L VB_12_) under ambient temperature and natural light. The MC-producing cyanobacteria *Microcystis aeruginosa* (strain FACHB-905, provided by the Institute of Hydrobiology, Chinese Academy of Sciences, Wuhan, China) was grown in BG-11 medium at 25 ± 1 °C under illumination at ca. 36 µE·(m^2^·s)^−1^ and a 14 h:10 h light/dark cycle. The green microalga *C. vulgaris* was a high nutritional food in aquaculture, so it was used as the control algae in this study [70].

### 5.2. Experimental Design and Sample Collection

Prior to the feeding exposure test, the snails were exposed to aerated tap water and fed with *C. vulgaris* once per day for 1 week (water hardness: 75 mg·L^−1^ calcium carbonate, light/dark cycle of 8 h:16 h, and temperature of 24 ± 0.5 °C). After 7 days of acclimation, the snails were randomly divided into two groups: treatment group (exposed to toxic *M. aeruginosa*, denoted as T group) and control group (exposed to nontoxic *C. vulgaris*, denoted as G group), with six replicates (equivalent to six glass containers) of each treatment. The experiment was conducted in 12 containers (34 cm × 19 cm × 22 cm), each initially filled with 12 L algal suspension and 40 snails. The algal density of *M. aeruginosa* was set as 1.8 × 10^7^ cells·mL^−1^, which was close to the peak density during cyanobacterial blooms, and the corresponding density of *C. vulgaris* was 2.5 × 10^6^ cells·mL^−1^ [71]. Full water renewal and algae additions were performed twice daily (09:00 and 21:00). As the number of snails decreased, the algal suspension was reduced accordingly to maintain 300 mL of algal suspension per snail. The experimental conditions during the exposure period were consistent with those during the acclimation period.

The snails were exposed to the above conditions for 14 days. On days 1, 3, 7, and 14, six snails were collected from every two containers and pooled into one replicate for RNA extraction (*n* = 3), and two snails from each container were collected for MC determination (*n* = 6). These snails were immediately dissected, and their hepatopancreas was collected, weighed, and preserved at −80 °C. After 14 days of exposure, the intestines of three snails from each container were pooled for analysis of microbial community (*n* = 6). The snails were checked for health every 8 h, and the dead individuals were discarded immediately.

### 5.3. MC Determination

The MCs were extracted as described in previous studies [13]. All the samples were determined using ELISA with an ELISA Microcystin Plate Kit (Beacon Analytical Systems, Portland, ME, USA; with a detection limit of 0.1 μg/L). The kit could detect many kinds of MCs but could not differentiate between MC-LR and other variants. Therefore, the MCs concentration in this analysis was regarded as the equivalents of MC-LR. In addition, the matrix effect and the recovery of MC extraction were determined, and they were consistent with previous results [30]. The average extraction efficiency was 91.6%, and the matrix effect was almost negligible due to small differences between methanol and the matrix results, with an average of 4.3%. The concentration of MCs in this research was represented by actual measured data owing to the high recovery efficiency and the low matrix effect.

### 5.4. Transcriptomic Analysis of Hepatopancreas

The total RNA from hepatopancreatic tissues was extracted using the TRIzol reagent (Vazyme, Nanjing, China). Each RNA sample was divided into two: one for RNA-seq and the other one for qRT-PCR. RNA purity and integrity were assessed using the NanoDrop 2000 (Thermo Scientific, Waltham, MA, USA) and Agilent 2100 bioanalyzer (Agilent, Santa Clara, CA, USA). A total of 24 RNA samples and a pooled sample from 24 RNA samples were sent to the sequencing facility for differential gene expression analysis and de novo transcriptomic analysis, respectively. For library construction, after DNA contamination was excluded with DNase I, the resultant RNAs were enriched using magnetic Oligo (dT) beads. Then, the mRNA was fragmented randomly and reverse transcribed into cDNA using an N6 primer. Afterwards, the cDNA was purified and ligated to adaptor sequences, and PCR amplification was used to obtain double-strand libraries. De novo transcriptome was carried out with 150 bp paired-end (PE) sequencing method, and differential expression analysis was sequenced by 50 bp single-end (SE) sequencing method using the BGISeq-500 platform (BGI, Wuhan, China). These raw data are freely available in the NCBI’s Sequence Read Archive (SRA) (SAMN32307618-SAMN32307641).

All raw reads were processed by discarding reads with adaptors, reads with unknown bases (>5%), and low-quality reads (>20% of low-quality bases), using SOAPnuke (v. 1.5.2) to obtain clean reads. De novo transcriptome assembly of *B. aeruginosa* was performed using Trinity (v. 2.8.4), and TGICL was used to cluster the assembled transcripts to obtain a single set of non-redundant genes. These genes were used as the reference transcriptome. Then, all genes were functionally aligned against the public databases (NR, NT, SwissProt, KOG, Pfam, and KEGG) using the software BLAST (v. 2.2.23) with the E-value cutoff of 10^−5^. GO annotation was performed by Blast2GO (v. 2.5.0) with the E-value cutoff of 10^−5^ based on the NR database.

The clean reads of each sample were mapped against the reference transcriptome of this study using Bowtie2 (v. 2.2.5) by default parameters. Gene expression was calculated using RSEM (v. 1.2.12) and normalized using fragments-per-kilobase-per-million-mapped-reads method. DESeq2 R package (v. 1.16.1) was used to screen DEGs with fold change >2 and false discovery rate (FDR) < 0.05. The R package ClusterProfiler was used to analyze the function enrichment of DEGs in KEGG. The GO enrichment analysis of DEGs was used to reveal the biological functions using the TopGO software (v. 2.42.0). The significantly enriched KEGG pathways and GO terms were defined with *p* value < 0.05. A Venn diagram was used to quantify the number of unique and shared DEGs in multiple timepoints. PCA-based all genes expression was then carried out to obtain an overall classification of samples using SIMCA-P software (v. 13.0.0.0, Umetrics AB, Umeå, Sweden).

### 5.5. qRT-PCR Validation

Six DEGs were randomly selected and detected by qRT-PCR to validate the transcriptomic results. Reverse transcription of RNA was conducted using the HiScript^®^ II Q RT SuperMix kit for Qpcr (Vazyme, Nanjing, China). qRT-PCR was performed using the Taq Pro Universal SYBR qPCR Master Mix kit (Vazyme, Nanjing, China) on an ABI 7500 real-time PCR system (Applied Biosystems, Waltham, MA) in accordance with the manufacturer’s instructions. Ribosomal protein L7 (*RPL7*) was selected as an internal reference gene. The primer sequences are listed in Table S6. The relative expression levels of RPL7 in the samples were calculated utilizing the 2^−ΔΔCt^ method. Each sample had three technical replicates.

### 5.6. DNA Extraction, Amplification, and Illumina Hiseq

The methods of DNA extraction, amplification, and library construction sequencing refer to the previous studies [30]. Unfortunately, one of the samples did not meet the criteria for construction of a sequencing library, so there were five biological replicates per treatment. The libraries were sequenced with the Illumina Hiseq2500 platform, and 250 bp PE reads were generated. The detailed information for the processing of raw sequencing data was the same as that in previous studies [30]. Raw data were deposited in the NCBI’s SRA (SAMN32305968-SAMN32305977).

### 5.7. Correlation Analysis of Gut Microbiota and DEGs

Spearman’s correlation analysis was performed to explore the correlations between gut microbiota and host stress response, immunity, and metabolism-related DEGs. The correlations and significant differences between gut microbiota and DEGs were shown in a heatmap.

### 5.8. Statistical and Bioinformatic Analysis

The data of daily mortality and MC concentration were represented as the mean ± SD. The normality of datasets was determined using SPSS Explore and Descriptive functions. Student’s *t*-test was used to determine the significant difference in daily mortality between two groups. Differences in MC concentrations at different timepoints within the same treatment group were analyzed by one-way ANOVA followed by Duncan’s multiple range test. Statistical analysis was performed using SPSS 23.0 (SPSS, Chicago, IL, USA), and values were considered significantly different when *p* value < 0.05.

For intestinal microbiota analysis, alpha diversity analyses, including Shannon, Simpson, Chao1, and Ace indices, were determined to assess bacterial diversity and richness. Student’s *t*-test was performed to identify between-group differences of alpha diversity indices. ANOSIM was performed to assess between-group differences. PCoA and HCA were conducted to examine intrinsic clusters and variations. PERMANOVA was used to test within- and between- group variations. Wilcoxon’s rank sum test was performed to identify significant differential abundance between two groups. PICRUSt2 analysis was used based on the KEGG database to further understand functional differences between two groups. Levels 2 and 3 of KEGG pathways were compared between the two groups based on *t*-test (*p* < 0.05). Network analysis was used to assess microbiome complexity for two groups. More than 0.02% of bacterial relative abundance was selected to avoid spurious correlations. Pearson’s correlations were calculated, and |r| >  0.70 and FDR < 0.01 were considered statistically robust and included to generate the bacterial networks using Gephi (v. 0.9.6).

## Figures and Tables

**Table 1:** List of differentially expressed genes in the hepatopancreas in response to toxic cyanobacteria.

Gene ID ^a^	NR Annotation	KO Name	log2 (Fold Change) ^b^			
			1 d	3 d	7 d	14 d
Oxidative response/xenobiotics biodegradation						
Unigene33914	thioredoxin-like protein 4B [*Pomacea canaliculata*]	*TXNL4A*	1.18 *	−0.54	1.51 **	−0.03
CL3662.Contig2	cytochrome P450 3A43-like [*Pomacea canaliculata*]	*CYP3A*	0.79	0.85	1.54 *	1.20 **
CL3025.Contig1	catalase-like isoform X1 [*Pomacea canaliculata*]	*CAT*	0.22	1.60 **	1.06 *	0.33
Unigene25071	sulfotransferase 1A3-like isoform X2 [*Pomacea canaliculata*]	*SULT1*	−0.38	1.18	1.24 **	1.78 **
CL9050.Contig1	pi-class glutathione S-transferase [*Cipangopaludina cathayensis*]	*GST*	0.81	−0.46	1.14 *	1.55 **
CL515.Contig1	glutathione peroxidase-like [*Pomacea canaliculata*]	*GPX4*	0.92	−0.14	1.13 *	1.25 **
CL5413.Contig1	heat shock protein 70 [*Mytilus coruscus*]	*HSPA1s*	−0.36	1.06 **	1.19 **	0.11
CL8943.Contig2	reactive oxygen species modulator 1-like isoform X1 [*Anneissia japonica*]	*ROMO1*	1.31 **	-0.44	−1.09 **	−0.24
Immune responses						
CL7726.Contig2	interleukin-6 receptor subunit beta-like isoform X2 [*Pomacea canaliculata*]	*IL6R*	1.13 **	0.31	2.31 **	1.03
Unigene58383	TNF receptor-associated factor 2-like isoform X1 [*Pomacea canaliculata*]	*TRAF2*	1.02 *	-0.62	1.11 *	0.26
Unigene43614	Toll-like receptor 8 [*Pomacea canaliculata*]	*TLR3*	1.40 *	-0.33	1.34 *	0.38
CL10774.Contig2	C-type lectin domain family 4 member M-like [*Pomacea canaliculata*]	*CLEC17A*	−0.79	1.16 *	1.24 *	0.84
CL10094.Contig1	tumor necrosis factor receptor superfamily member 13B-like [*Pomacea canaliculata*]	*TNFRSF13B*	2.01 **	1.93 **	1.66	0.24
CL1261.Contig2	caspase-3-like [*Pomacea canaliculata*]	*CASP7*	−0.32	0.71	1.54 **	1.15 *
CL4333.Contig1	apoptosis-inducing factor 1, mitochondrial-like isoform X3 [*Pomacea canaliculata*]	*AIFM1*	1.71 *	0.38	0.13	1.52 *
Unigene46579	apoptosis inhibitor 5-like [*Pomacea canaliculata*]	*API5*	1.62 *	1.03 *	-0.15	-0.81
Unigene44570	complement C1q-like protein 3 [*Pomacea canaliculata*]	*C1qL3*	−0.53	1.16 **	1.06	1.61 **
Unigene56138	complement C1q-like protein 2 [*Pomacea canaliculata*]	*C1qL2*	−1.30 *	0.29	4.01 **	1.19
Energy metabolism						
Unigene9677	aconitate hydratase, mitochondrial-like [*Aplysia californica*]	*ACO*	−0.84	1.20 *	−1.68 **	-
Unigene53307	ATP-citrate synthase-like isoform X2 [*Pomacea canaliculata*]	*ACLY*	1.13 *	-0.07	1.06 *	0.75
CL11869.Contig1	hexokinase type 2-like [*Pomacea canaliculata*]	*HK*	2.39 **	-	1.02	2.10 **
CL4947.Contig1	aldehyde dehydrogenase family 3 member B1-like isoform X5 [*Pomacea canaliculata*]	*ALDH*	−0.73	1.88 *	1.09 *	0.49
Unigene44545	pyruvate kinase PKM-like isoform X4 [*Pomacea canaliculata*]	*PK*	2.01 **	0.16	1.09 **	0.53
Unigene61388	aldose 1-epimerase-like [*Pomacea canaliculata*]	*GALM*	1.56	−1.78 **	0.24	1.84 *
CL10453.Contig2	NADH dehydrogenase [ubiquinone] 1 alpha subcomplex subunit 5-like [*Pomacea canaliculata*]	*NDUFA5*	−1.52	7.35 **	1.30 **	1.63
Unigene63153	pancreatic triacylglycerol lipase-like [*Pomacea canaliculata*]	*PNLIP*	0.34	0.61	1.69 *	1.26 *
Unigene34763	peroxisomal acyl-coenzyme A oxidase 1-like [*Pomacea canaliculata*]	*ACOX1*	1.57 *	0.01	1.56 **	0.47
Unigene56283	peroxisomal acyl-coenzyme A oxidase 3-like [*Pomacea canaliculata*]	*ACOX3*	1.02 *	-0.01	1.17 *	0.94
CL10117.Contig4	fatty acid desaturase 2-like isoform X1 [*Pomacea canaliculata*]	*FADS2*	2.00 **	1.13	3.11 **	0.52
CL8795.Contig2	acetyl-CoA acetyltransferase, mitochondrial-like [*Pomacea canaliculata*]	*ACAT*	−1.21 *	1.91 **	1.43	0.07
Unigene43422	carnitine O-palmitoyltransferase 1, liver isoform-like isoform X2 [*Pomacea canaliculata*]	*CPT1A*	2.14 **	1.24 *	0.63	0.15
CL6049.Contig1	glutamine synthetase-like [*Pomacea canaliculata*]	*glnA*	1.34 *	1.65 *	1.06	0.46
CL2579.Contig1	aminoacylase-1-like [*Pomacea canaliculata*]	*ACY1*	1.39 *	0.10	1.08 *	0.23
CL5226.Contig1	carnitine O-acetyltransferase-like isoform X1 [*Pomacea canaliculata*]	*CRAT*	0.83	1.51 *	0.33	1.66 *
CL3812.Contig4	N-alpha-acetyltransferase 20-like [*Pomacea canaliculata*]	*NAA20*	0.34	2.07 *	1.63 *	0.85
Unigene26308	methionine aminopeptidase 1-like isoform X1 [*Pecten maximus*]	*MAP1*	−0.85	1.33 *	−0.26	1.44 *

^a^ More information on DEGs are listed in Table S2. ^b^ The fold changes are indicated as compared with the control group. Genes with |log2 (fold change)| > 1 and FDR < 0.05 were considered as differentially expressed genes (* FDR < 0.05; ** FDR < 0.01).

**Table 2:** Network parameters.

Index	G	T
node	192	289
edge	617	1467
avgK	6.427	10.152
avgCC	0.72	0.733
positive/negative (%)	90.11/9.89	85.55/14.45

## Data Availability

The high-throughput sequence data of this study have been deposited in the NCBI Sequence Read Archive under accession numbers PRJNA913809 and PRJNA913703. All other data are presented in the manuscript or Supplementary Materials.

## Supplementary Materials

The following supporting information can be downloaded at: https://www.mdpi.com/article/10.3390/toxins15020119/s1, Table S1: Overview of 24 samples transcriptome sequencing data; Table S2: All differentially expressed genes in the hepatopancreas of *Bellamya aeruginosa* after exposure to toxic cyanobacteria; Table S3: Relative abundance of gut microbiota affected by exposure to toxic cyanobacteria at the phylum level; Table S4: Relative abundance of gut microbiota affected by exposure to toxic cyanobacteria at the genus level; Table S5: The abundance ratio of gut microbiota between the G group and the T group predicted the level 3 function; Table S6: The primers used in the present study; Figure S1: GO classification of DEGs; Figure S2: The numbers of shared and unique OTUs between the G group and T group by the Venn diagram; Figure S3: Analysis of similarity (ANOSIM) of gut microbiota; Figure S4: Correlation network based on the bacteria phylum.
