# Supplementary material for: Integration of Transcriptomics and Microbiomics Reveals the Responses of Bellamya aeruginosa to Toxic Cyanobacteria
Source: Toxins (Basel). 2023 Feb 1;15(2):119. doi: 10.3390/toxins15020119 (PMC9958990; doi:10.3390/toxins15020119)
Supplement: Supplementary file 1 [file toxins-15-00119-s001.zip › toxins-2158149 supplementary figures.pdf]

toxins

Article

Integration of Transcriptomics and Microbiomics Reveals the Responses of *Bellamya aeruginosa* to Toxic Cyanobacteria

Xianming Yang, Jinyong Zhu, Chaoyang Hu, Wen Yang and Zhongming Zheng

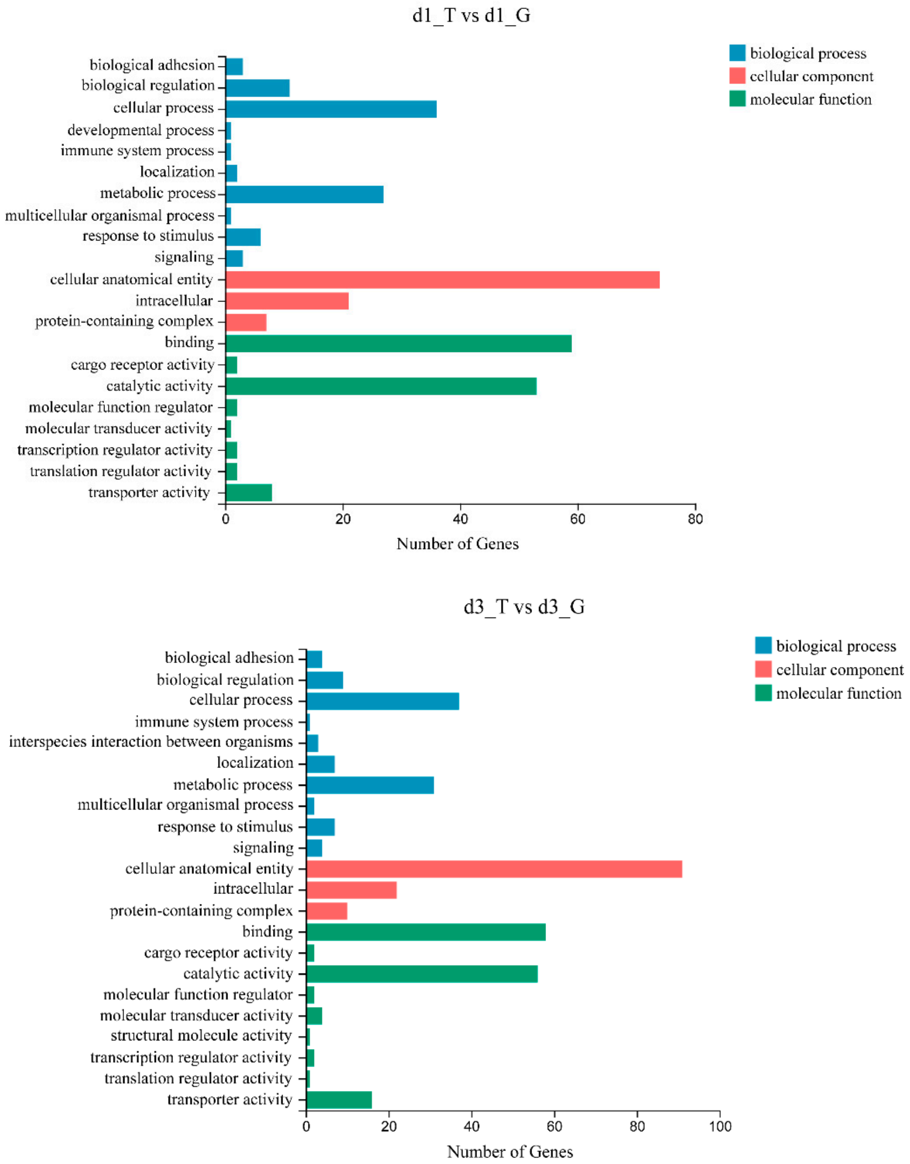

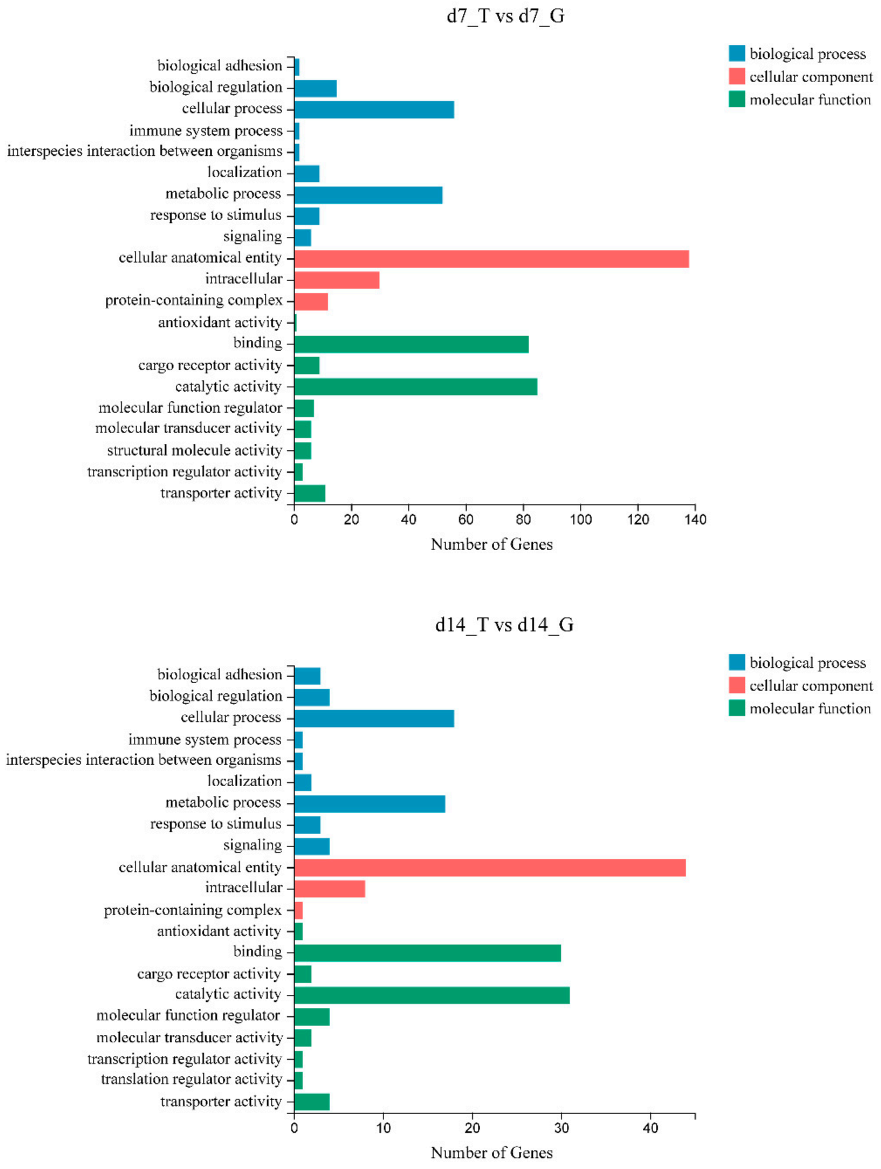

Figure S1. GO classification of DEGs.

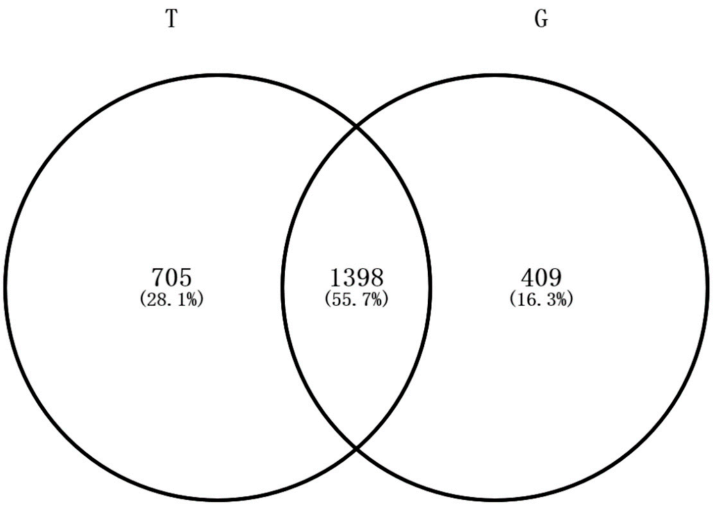

Figure S2. The numbers of shared and unique OTUs between the G group and T group by the Venn diagram.

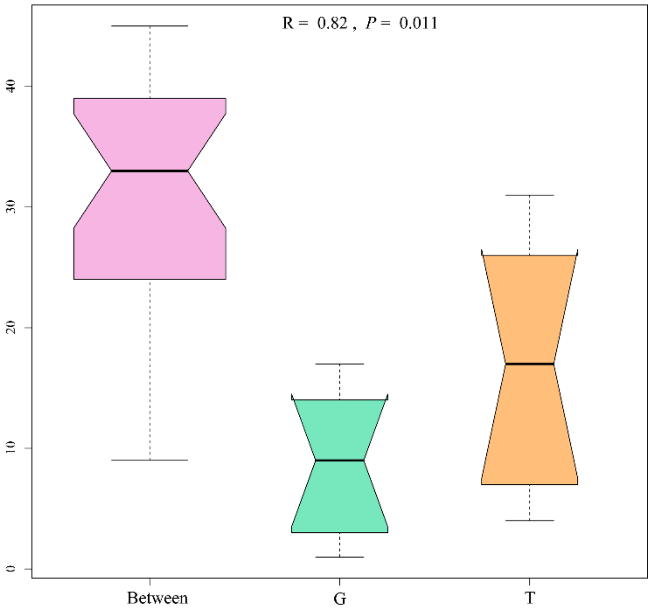

Figure S3. Analysis of similarity (ANOSIM) of gut microbiota.

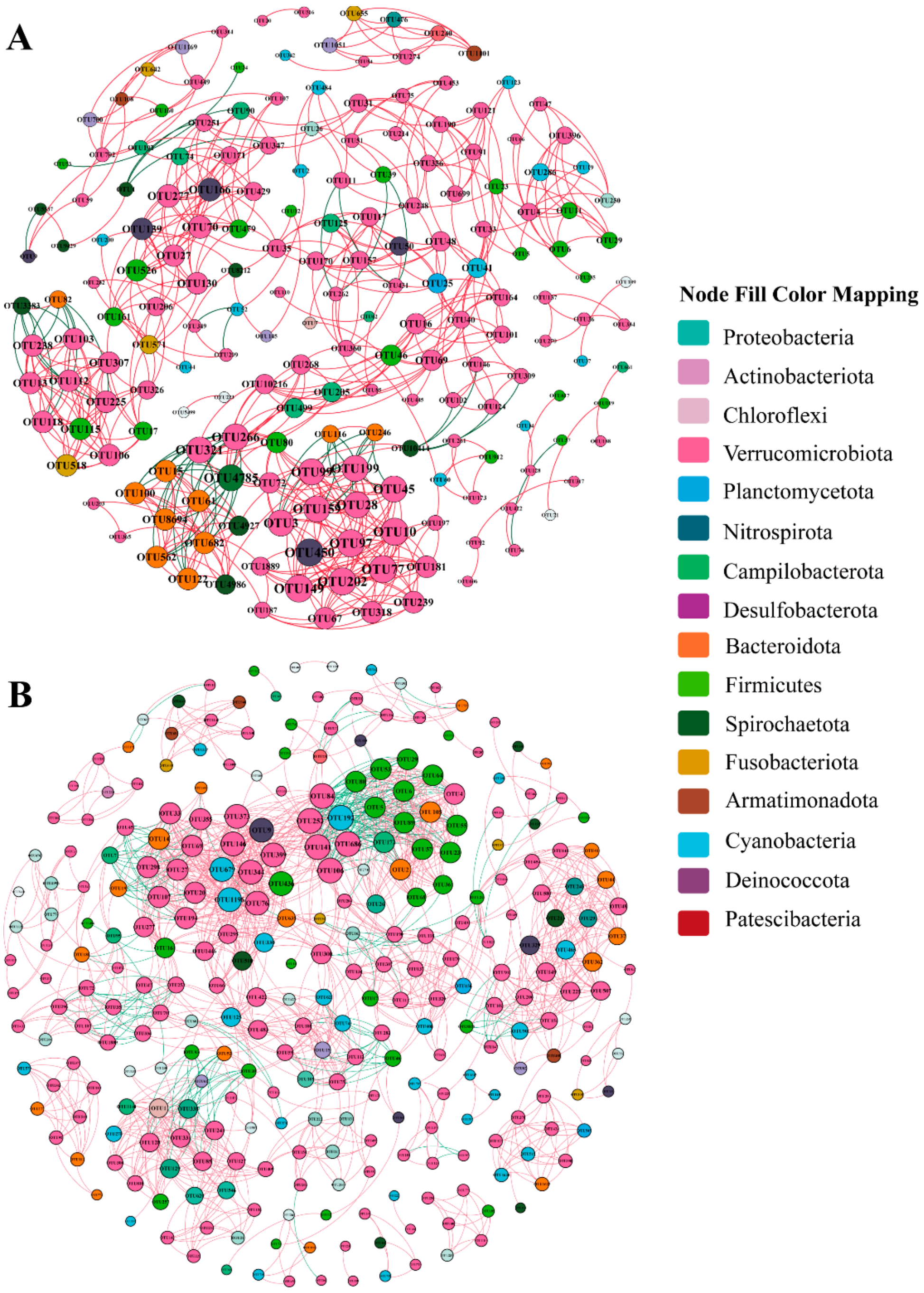

Figure S4. Correlation network based on the bacteria phylum. (A) G group. (B) T group. The circle nodes represent the bacteria taxa, and the size indicates its relative abundance. Green lines indicate a negative correlation, and red lines represent a positive correlation.
